# Increased Histological Tumor Pigmentation in Uveal Melanoma Is Related to Eye Color and Loss of Chromosome 3/BAP1

**DOI:** 10.1016/j.xops.2023.100297

**Published:** 2023-03-11

**Authors:** Maria Chiara Gelmi, Annemijn P.A. Wierenga, Wilma G.M. Kroes, Sjoerd G. van Duinen, Jessica S. Karuntu, Marina Marinkovic, Jaco C. Bleeker, Gregorius P.M. Luyten, T.H. Khanh Vu, Robert M. Verdijk, Martine J. Jager

**Affiliations:** 1 Department of Ophthalmology, Leiden University Medical Center, Leiden, The Netherlands; 2 Department of Clinical Genetics, Leiden University Medical Center, Leiden, The Netherlands; 3 Department of Pathology, Leiden University Medical Center, Leiden, The Netherlands; 4 Section Ophthalmic Pathology, Department of Pathology, Erasmus MC University Medical Center, Rotterdam, The Netherlands

**Keywords:** Eye, Melanoma, Oncology, Pigmentation, Prognosis

## Abstract

**Purpose:**

Heavy pigmentation is known to be a prognostic risk factor in uveal melanoma (UM). We analyzed whether genetic tumor parameters were associated with tumor pigmentation and whether pigmentation should be included in prognostic tests.

**Design:**

Retrospective comparison of clinical, histopathological, and genetic features and survival in UM with different pigmentation.

**Participants:**

A total of 1058 patients with UM from a White European population with diverse eye colors enucleated between 1972 and 2021.

**Methods:**

Cox regression and log-rank tests were used for survival analysis; the chi-square test and Mann–Whitney *U* test were used for correlation analysis.

**Main Outcome Measures:**

Uveal melanoma–related survival based on tumor pigmentation and chromosome status, correlation of tumor pigmentation with prognostic factors.

**Results:**

The 5-year UM-related mortality was 8% in patients with nonpigmented tumors (n = 54), 25% with lightly pigmented tumors (n = 489), 41% with moderately pigmented tumors (n = 333), and 33% with dark tumors (n = 178) (*P* < 0.001). The percentage of tumors with monosomy 3 (M3) or 8q gain increased with increasing pigmentation (31%, 46%, 62%, and 70% having M3 [*P* < 0.001], and 19%, 43%, 61%, and 63% having 8q gain [*P* < 0.001] in the 4 increasing pigment groups, respectively). BRCA-associated protein 1 **(**BAP1) loss (known for 204 cases) was associated with increased tumor pigmentation (*P* = 0.001). Cox regression analysis on survival showed that when chromosome status and pigmentation were both included, pigmentation was not an independent prognostic indicator. Preferentially expressed antigen in melanoma (PRAME) expression was a significant prognostic marker in light tumors (*P* = 0.02) but not in dark tumors (*P* = 0.85).

**Conclusions:**

Patients with moderately and heavily pigmented tumors showed a significantly higher UM-related mortality than patients with unpigmented and light tumors (*P* < 0.001), supporting prior reports on the relation between increased tumor pigmentation and a worse prognosis. Although we previously showed that a dark eye color was associated with tumor pigmentation, we now show that the tumor’s genetic status (chromosome 3 and 8q/BAP1 status) is also related to tumor pigmentation. When pigmentation and chromosome 3 status are both included in a Cox regression analysis, pigmentation is not an independent prognostic factor. However, evidence from this and previous studies shows that chromosome changes and PRAME expression have a stronger association with survival when they occur in light tumors than in dark ones. *F****inancial Disclosure(s)***: Proprietary or commercial disclosure may be found after the references.

Uveal melanoma (UM) is the most common primary intraocular malignancy in adults; it develops most frequently in the choroid (90%) and less frequently in the ciliary body (6%) and iris (4%).^1^ Uveal melanoma originates from melanocytes in the uvea, which are neural crest-derived cells that contain melanosomes and give rise to pigmentation. Uveal melanomas vary in their level of pigmentation, both macroscopically and microscopically, from completely amelanotic to heavily pigmented; some tumors show areas with different degrees of pigmentation, which can even be visualized by magnetic resonance imaging.^2^^,^^3^

The degree of tumor pigmentation is part of the standard histopathological analyses of UM, and several reports show an association between heavy pigmentation and a poor prognosis. McLean et al^4^ studied small choroidal and ciliary body melanomas and reported that a high degree of microscopic pigmentation constituted a negative prognostic factor; they suggested that this prognostic significance was related to the higher number of epithelioid cells in dark tumors. Packard et al^5^ studied choroidal melanomas and reported heavy pigmentation to be a poor prognostic factor, especially in spindle cell tumors. Seddon et al^6^ showed that tumor pigmentation was one of the 5 factors that best predicted prognosis (the other 4 being the presence of epithelioid cells, tumor diameter, location of the anterior tumor margin, and invasion of the transection line). Similarly, the Collaborative Ocular Melanoma Study reported that UM with heavy microscopic pigmentation tended to be larger and contained more epithelioid cells than tumors with little pigmentation.^7^ Regan et al^8^ studied eyes with UM treated with proton beam radiation and showed that both blue eye color and heavy clinical pigmentation were associated with a worse survival and that the risk of death was even higher in patients with blue eyes and dark tumors. More recently, the Shields’ group analyzed a cohort of 8100 cases and concluded that heavy macroscopic (clinical) pigmentation was a negative prognostic factor.^9^ Markiewicz et al^10^ analyzed macroscopic (clinical) pigmentation as well and compared 26 amelanotic and 128 pigmented UMs. Their findings agree with previous studies in terms of survival, with the added report of a higher proportion of BRCA-associated protein 1 (BAP1) negative cases among the pigmented tumors.

Several of these reports mention that increased pigmentation was associated with the presence of epithelioid cells, which is known to be associated with chromosome 3 loss, one of the strongest risk factors for developing metastases.^11^^,^^12^ The loss of one chromosome 3 is very often accompanied by a mutation in the *BAP1* gene on the other chromosome 3, leading to loss of BAP1 protein expression.^13^, ^14^, ^15^

We recently showed that eye color influences tumor pigmentation, with light eyes having less pigmented tumors than dark eyes,^16^ confirming the observation reported by Regan et al.^8^ Furthermore, monosomy 3 (M3) had a greater influence on survival in patients with light eyes than in those with dark eyes.

We wondered whether tumor pigmentation is an independent prognostic factor for survival in patients with a UM and whether tumor pigmentation is not only associated to the genetically determined iris color but also related to the tumor’s genetic status. Therefore, we set out to compare histopathologic tumor characteristics, chromosome 3 and 8q status, and BAP1 expression in tumors with different pigmentation levels. We also looked at the relation between these genetic features and survival in patients with UM with different degrees of histological tumor pigmentation.

## Methods

### Approval

This project adhered to Dutch law and the tenets of the Declaration of Helsinki (World Medical Association of Declaration 1964; ethical principles for medical research involving human subjects). Materials and histopathological, genetic, and follow-up data are part of Biobank OOG-2 of the Leiden University Medical Center (Uveamelanoomlab-2019-7, approval: May 2019). Permission was given to use these data for this analysis by the medical ethics committee (no.: B20.022).

### Patients

We performed a retrospective cohort study of patients with UM enucleated at the Leiden University Medical Center between August 1972 and October 2021 and selected 1197 cases in which tumor pigmentation had been recorded. We excluded patients who had received some form of irradiation treatment before enucleation. The total number of analyzed cases was 1058. Tumor pigmentation was scored macroscopically by the operator during dissection of the eye in 2 halves, as per standard preparation for pathological analysis. The pigmentation was scored on a 4-point scale as follows: 1—unpigmented (white), 2—lightly pigmented (gray), 3—moderately pigmented (brown), and 4—heavily pigmented (dark brown–black). Examples of tumors in each pigmentation group can be found in Fig S1. In the case of tumors with heterogenous pigmentation, the pigmentation of the most prominent component was scored. When only 2 groups of tumor pigmentation were used for analysis, groups 1 and 2 were classified as light, and groups 3 and 4 were classified as dark. Cell type, ciliary body involvement, episcleral growth, and tumor diameter and thickness were evaluated during the histopathological examination performed by 1 of 3 experienced pathologists. The American Joint Committee on Cancer stage was scored according to the eighth edition of the American Joint Committee on Cancer staging manual.^17^^,^^18^ Chromosome copy number was determined either by karyotyping, fluorescence in situ hybridization, or single nucleotide polymorphism (SNP) array, and when any of these tests showed an abnormality, the case was considered aberrant (M3 or chromosome 8q gain).^16^ BAP1 staining was performed with immunohistochemistry as previously described^14^^,^^15^ and scored by an ophthalmic pathologist. Clinical and survival information was collected from patient charts, and follow-up time was defined as the time from enucleation to death or last recorded patient contact. Information on death and cause of death was obtained from the Integral Cancer Center West. The median follow-up of this population was 57 months (range: 0–547 months, mean: 110).

Eye color information was available for 412 cases, and it was collected from medical charts, clinical photographs, and self-reported iris color as previously described.^16^ Iris color was scored as blue/gray, green/hazel, and dark brown.

### Preferentially Expressed Antigen in Melanoma mRNA Expression

Preferentially expressed antigen in melanoma (PRAME) expression was available for 64 cases. messenger RNA expression was measured from archived snap-frozen material on an Illumina HT-12v4 chip (Illumina) using probe ILMN_1700031. Preferentially expressed antigen in melanoma expression was classified as positive or negative according to a cutoff value set at the inflection point of the *PRAME* expression curve obtained using probe ILMN_1700031.^19^

### Statistical Analysis

Statistical analyses were performed with the statistical software SPSS (IBM SPSS Statistics for Windows, version 25.0; IBM Corp). Survival was evaluated with a Kaplan–Meier curve and log-rank test and with Cox regression, correcting for age and sex. In both cases, patients who died with UM metastases were considered as events, and patients who were alive at the end of follow-up, lost to follow-up, or dead because of other causes were censored. Categorical variables were compared with the Pearson chi-square test, whereas continuous variables were compared with the Mann–Whitney *U* test or Kruskal–Wallis test in analyses with > 2 groups. Logistic regression was performed with tumor pigmentation as a binary dependent variable (light or dark) and eye color and chromosome 3 status as categorical independent variables. A *P* value < 0.05 was considered significant. In addition, Bonferroni correction was applied in tables with multiple comparisons Tables S8, S9, and the adjusted α is reported in the footnote of each table. Because of the strong association between variables tested, we deemed a multivariable analysis not to be an ideal method to reduce multiple testing bias in this case.

## Results

The Leiden UM enucleation database includes all UM cases who have been enucleated for UM at the Leiden University Medical Center from 1972 to 2021. Of these 1291 UM cases, information regarding macroscopic tumor pigmentation at the time of enucleation was available for 1197 cases. Because irradiation might influence tumor features, we excluded UM cases who had undergone treatment such as radioactive plaque irradiation or proton beam therapy before enucleation. This left 1058 cases for our study.

Based on macroscopic analysis of enucleated UM, tumors were assigned to 1 of 4 pigmentation categories. Of the 1058 cases in our cohort, 55 (5%) were classified as unpigmented, 489 (46%) as lightly pigmented, 336 (32%) as moderately pigmented, and 178 (17%) as heavily pigmented. We first set out to compare pigmentation with UM-related mortality to determine whether the level of pigmentation had prognostic value in our European patient group. Four cases did not have follow-up data and were excluded from survival analyses.

**Figure 2:**
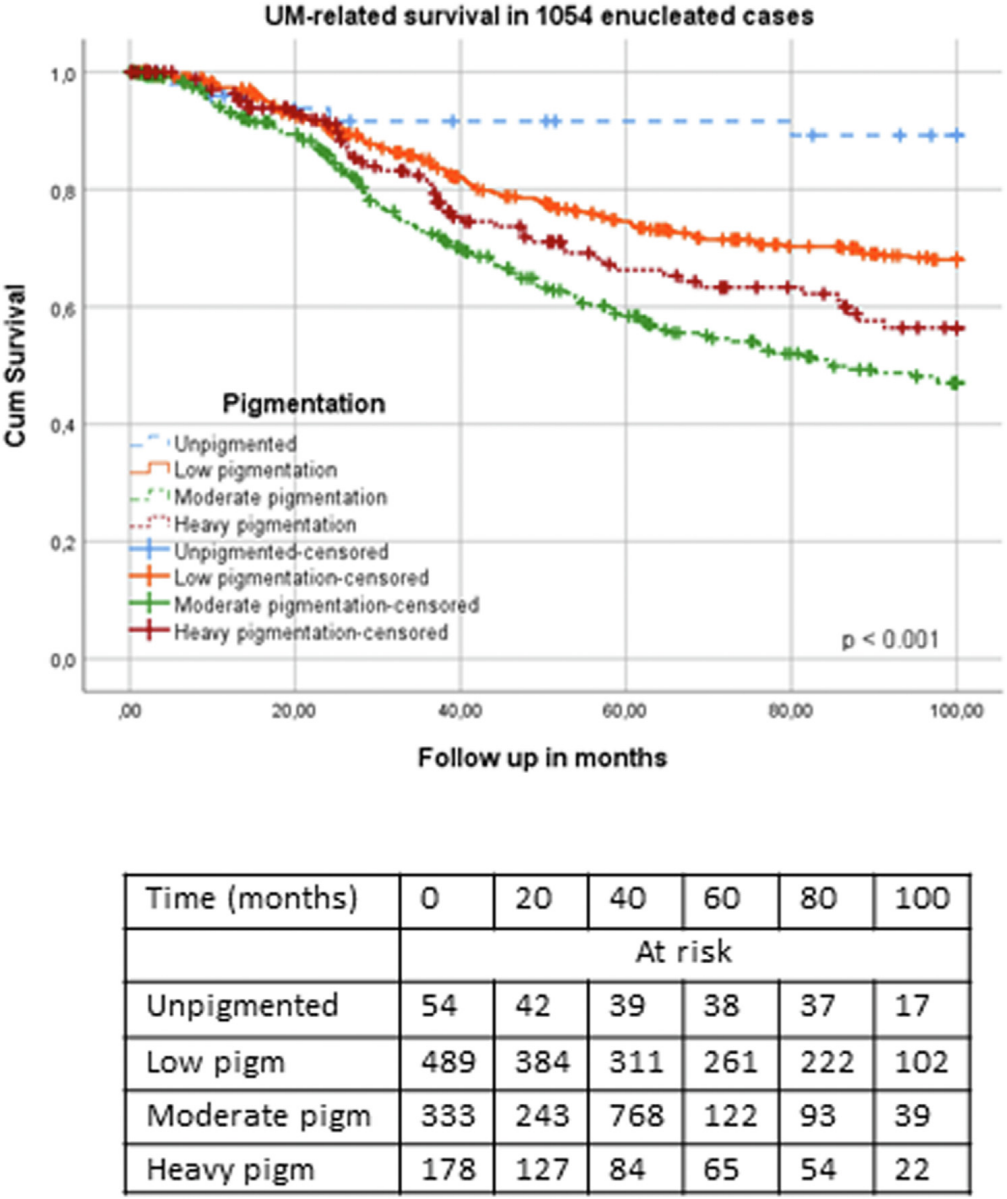
Survival in 1054 UM enucleated cases with different degrees of tumor pigmentation. Kaplan–Meier curve with log-rank test (*P* < 0.001). UM = uveal melanoma.

Patients with moderately and heavily pigmented tumors showed a significantly worse UM-related overall survival than patients with unpigmented and light tumors (Fig 2 and Table 1) (*P* < 0.001, both for Kaplan–Meier curves and Cox regression). Table S2 shows the results of Cox regression analysis comparing dark and light tumors (*P* < 0.001, Table S3). The difference in survival between the moderate and heavy pigmentation groups was not significant (Cox regression: *P* = 0.09, Table S3).

**Table 1:** Tumor Pigmentation and Chromosome 3 and 8q Status vs. UM-Related Survival in 1054 Patients with UM. Cox Regression for Effect of Macroscopic Histological Pigmentation (4 Groups) on UM-Related Survival, Correcting for Age and Sex in 1054 Patients with UM

	Wald	*P* Value	HR	CI
Pigmentation∗	31.301	**<0.001**		
Unpigmented†				
Low pigmentation	6.042	**0.014**	**3.067**	**1.255–7.498**
Moderate pigment	13.862	**<0.001**	**5.464**	**2.265–13.359**
Heavy pigmentation	9.245	**0.002**	**4.151**	**1.658–10.388**

Boldface indicates significant factors. CI = confidence interval; HR = hazard ratio; UM = uveal melanoma.

∗ Adjusted for age and sex.

† Reference group.

We then compared the clinical, histopathological, and genetic characteristics of the 4 pigmentation groups. As shown in Table 4, an increase in pigmentation correlated with an older age at diagnosis (*P* < 0.001), a higher frequency of occurrence of an epithelioid and mixed cell type (*P* = 0.001), more frequent ciliary body involvement (*P* < 0.001), a higher American Joint Committee on Cancer stage (*P* = 0.001), and a shorter median follow-up (*P* < 0.001). These findings show that our data agree with prior results, that is, that dark tumors more often show clinical and histopathological features associated with a poor prognosis. We subsequently compared genetic features: more pigment was associated with a higher proportion of tumors carrying M3 compared with disomy 3 (D3) (*P* < 0.001), chromosome 8q gain compared with normal 8q status (*P* < 0.001), and loss of BAP1 staining (*P* = 0.001) (Table 4). Therefore, we clearly demonstrate that, in addition to having adverse clinical and histopathological features, dark tumors more often show prognostically unfavorable chromosome aberrations.

**Table 4:** Comparison between Clinical and Histopathological and Genetic Features and Tumor Pigmentation (4 Groups) in 1058 Patients with UM

Feature	Unpigmented (55)§	Low Pigmentation (489)§	Moderate Pigmentation (336)§	Heavy Pigmentation (178)§	*P* Value¶
Gender					0.22∗
Male (575)	32 (58%)	249 (51%)	191 (57%)	103 (58%)	
Female (483)	23 (42%)	240 (49%)	145 (43%)	75 (42%)	
Age at enucleation (yrs)‖	56.08 (21.3–81.3)	60.67 (6.0–93.0)	65.19 (6.7–92.7)	64.12 (8.6–93.4)	**<0.001**†
Median follow-up (mos)‖	159.18 (1.4–547.3)	73.36 (0.4–542.8)	43.78 (0–542.3)	40.36 (0.1–542.3)	**<0.001**†
Largest basal diameter‖	11 (4–24)	11 (1–30)	12 (2–20)	12 (2–25)	**<0.001**†
Thickness‖	6 (1.5–17)	5.5 (0.5–15)	6 (0.8–15)	7 (0.8–17)	**0.003**†
Cell type					**0.001**∗
Spindle (352)	27 (49%)	182 (37%)	99 (30%)	44 (25%)	
Epithelioid or mixed (700)	28 (51%)	305 (63%)	263 (70%)	131 (75%)	
Ciliary body involvement					**<0.001**∗
No (735)	51 (93%)	365 (75%)	36 (64%)	104 (58%)	
Yes (323)	4 (7%)	124 (25%)	121 (36%)	74 (42%)	
Scleral ingrowth					0.73∗
None/superficial (677)	35 (65%)	315 (65%)	207 (63%)	120 (67%)	
Deep/total (370)	19 (35%)	169 (35%)	124 (36%)	58 (33%)	
AJCC					**0.001**∗
I–IIB (806)	47 (89%)	390 (84%)	243 (75%)	126 (73%)	
IIIA–IIIC (212)	6 (11%)	77 (17%)	83 (26%)	46 (27%)	
Chromosome 3 status					**<0.001**∗
Disomy (214)	11 (69%)	119 (54%)	56 (38%)	28 (30%)	
Monosomy (262)	5 (31%)	100 (46%)	92 (62%)	65 (70%)	
8q status					
Normal (211)	13 (81%)	116 (57%)	52 (39%)	30 (38%)	**<0.001**∗
Gain (223)	3 (19%)	88 (43%)	82 (61%)	50 (63%)	
BAP1 expression					**0.001**∗
BAP1 positive (79)	2 (40%)	50 (54%)	19 (29%)	8 (20%)	
BAP1 negative (125)	3 (60%)	43 (46%)	46 (71%)	33 (81%)	

Boldface indicates significant factors. AJCC = American Joint Committee on Cancer; BAP1 = BRCA-associated protein 1.

∗ Pearson chi-square test.

† Kruskal–Wallis test.

§ Percentages were calculated excluding missing data; percentages are rounded and may not total 100.

‖ Median (min–max).

¶ α after Bonferroni correction: 0.004.

### Association of Chromosome Status and Eye Color with Tumor Pigmentation

Although we now show a correlation between tumor and chromosome 3/BAP1 status and chromosome 8q (Table 4), we previously^16^ reported that eye color is also related to tumor pigmentation: patients with brown eyes more frequently had dark tumors than patients with blue or green eyes, who more often had light tumors. These findings confirm a previous report by Regan et al.^8^ To understand whether eye color and chromosome 3 and 8q status are independently associated to tumor pigmentation, we computed a logistic regression with tumor pigmentation as the binary dependent variable and eye color and chromosome 3 and 8q status as the independent variables. As Table 5 shows, both M3 and brown eye color were significant predictors of tumor pigmentation (D3 with 8q gain: *P* = 0.009, odds ratio [OR] = 3.138, confidence interval [CI] = 1.337–7.364, Wald = 6.902; M3: *P* < 0.001, OR = 3.229, CI = 1.855–5.620, Wald = 17.189; brown eye color: *P* = 0.002, OR = 3.271, CI = 1.524–7.016, Wald = 9.258).

**Table 5:** Logistic Regression Testing the Predictive Power of Eye Color and Chromosome 3 and 8q on the Level of Tumor Pigmentation (Light vs. Dark)†

	Wald	*P* Value	OR (CI)
Eye color	13.858	**0.001**	
Blue∗			
Green	2.479	0.12	0.597 (0.314–1.135)
Brown	9.258	**0.002**	3.271 (1.525–7.016)
Chromosome status	18.047	**< 0.001**	
D3, no 8q gain∗			
D3, 8q gain	6.902	**0.009**	3.138 (1.337–7.364)
M3	17.189	**< 0.001**	3.229 (1.855–5.620)

CI = confidence interval; D3 = disomy 3; M3 = monosomy 3; OR = odds ratio. Boldface indicates significant factors.

∗ Reference category.

† Light = unpigmented + low pigmentation; dark = moderate pigmentation + heavy pigmentation.

We decided to explore these associations further.

The chi-square test in Table 6 shows that M3 and 8q gain were especially related to increased tumor pigmentation in the blue eye group (*P* = 0.001), with a lower but still significant association in the brown eye group (*P* = 0.04) (Table 6).

**Table 6:** Distribution of Chromosome 3 Status and Chromosome 8q Status According to Tumor Pigmentation in 3 Groups with Different Eye Colors (*P* Value Determined by Chi-Square Test)

	D3, Normal 8q (92)	D3, 8q gain (32)	Monosomy 3 (164)	*P* Value
Blue eyes (189)				**0.001**∗
Light tumor (95)	42 (22%)	10 (5%)	43 (23%)	
Dark tumor (94)	18 (10%)	11 (6%)	65 (34%)	
Green eyes (54)				0.44†
Light tumor (34)	14 (26%)	3 (6%)	17 (32%)	
Dark tumor (20)	5 (9%)	3 (6%)	12 (22%)	
Brown eyes (45)				**0.044**†
Light tumor (11)	6 (13%)	0 (0%)	5 (11%)	
Dark tumor (34)	7 (26%)	5 (11%)	22(49%)	

Bold face indicates significant factors. CI = confidence interval; D3 = disomy 3; HR = hazard ratio; UM = uveal melanoma.

Eye color groups are analyzed separately, and percentages are calculated per eye color group.

∗ Chi-square test.

† Likelihood ratio.

### Is Pigmentation a Prognostic Factor Independent of Chromosome 3 Status?

To determine whether pigmentation was related to survival independently of chromosome 3 and 8q status, we used a Cox regression model (Table 7) that included both eye color and chromosome 3 and 8q status while correcting for age and sex. Once chromosome 3 and 8q status were taken into account, tumor pigmentation did show a significant association with survival.

**Table 7:** Tumor Pigmentation and Chromosome 3 and 8q Status vs. UM-Related Survival in 1054 Patients with UM. Cox Regression for Effect of Macroscopic Histological Pigmentation (2 Groups) and Chromosome 3 and 8q Status on UM-Related Survival, Correcting for Age and Sex in 1054 Patients with UM

	Wald	*P* Value	HR	CI
Dark vs. light pigmentation∗^,^†	0.008	0.93	0.984	0.692–1.398
Monosomy 3 vs. disomy 3∗	24.786	**<0.001**	**3.146**	**2.003–4.939**
8q gain vs. normal 8q	20.471	**<0.001**	**2.692**	**1.753–4.135**

Bold face indicates significant factors. CI = confidence interval; HR = hazard ratio; UM = uveal melanoma.

Eye color groups are analyzed separately, and percentages are calculated per eye color group. D3 = disomy 3.

∗ Adjusted for age and sex.

† Light = unpigmented + low pigmentation; dark = moderate pigmentation + heavy pigmentation.

**Figure 3:**
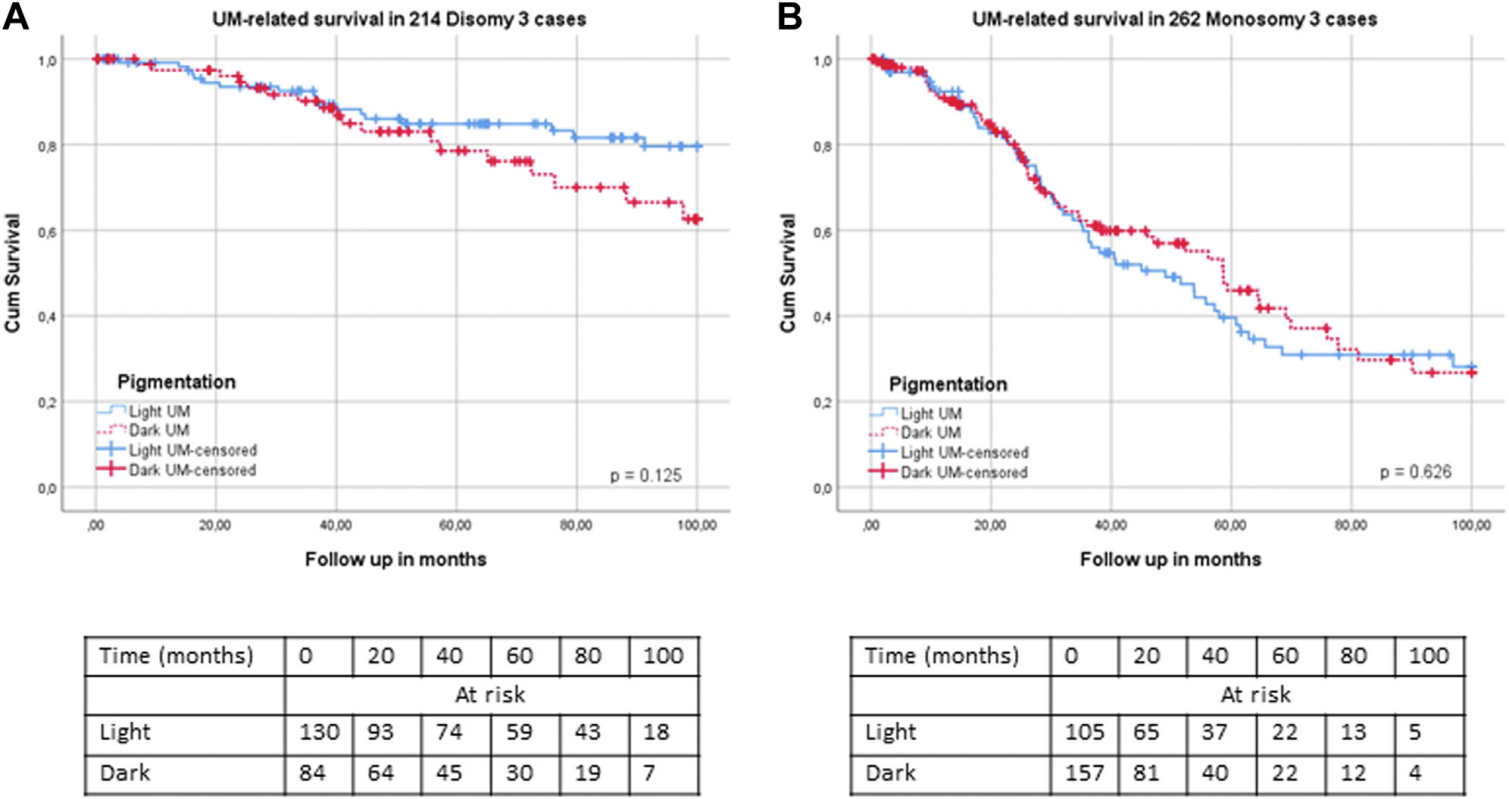
Survival in patients with UM with different levels of tumor pigmentation in disomy 3 and monosomy 3 groups. **A,** Uveal melanoma–related survival in 214 disomy 3 cases (*P* = 0.13). **B,** Uveal melanoma–related survival in 262 monosomy 3 cases (*P* = 0.63). UM = uveal melanoma.

This conclusion can also be reached by analyzing the relation between pigmentation and survival in patients with either D3 or M3 tumors separately (Fig 3). Within the D3 population, the curves for dark and light tumors diverge slightly at 5 years, although the difference does not reach significance (*P* = 0.13), whereas in the M3 group, the curves for light and dark tumors never diverge (*P* = 0.63).

We then compared the distribution of clinical and histopathological tumor features between light and dark tumors in the D3 and the M3 subcohorts separately. Among the D3 tumors, dark UM more frequently showed chromosome 8q gain (*P* = 0.003) (Table S8) compared with light UM, whereas in the M3 population, none of the factors analyzed showed a significant difference between light and dark UMs (Table S9).

### Influence of Pigmentation on Impact of Prognostic Factors

**Figure 4:**
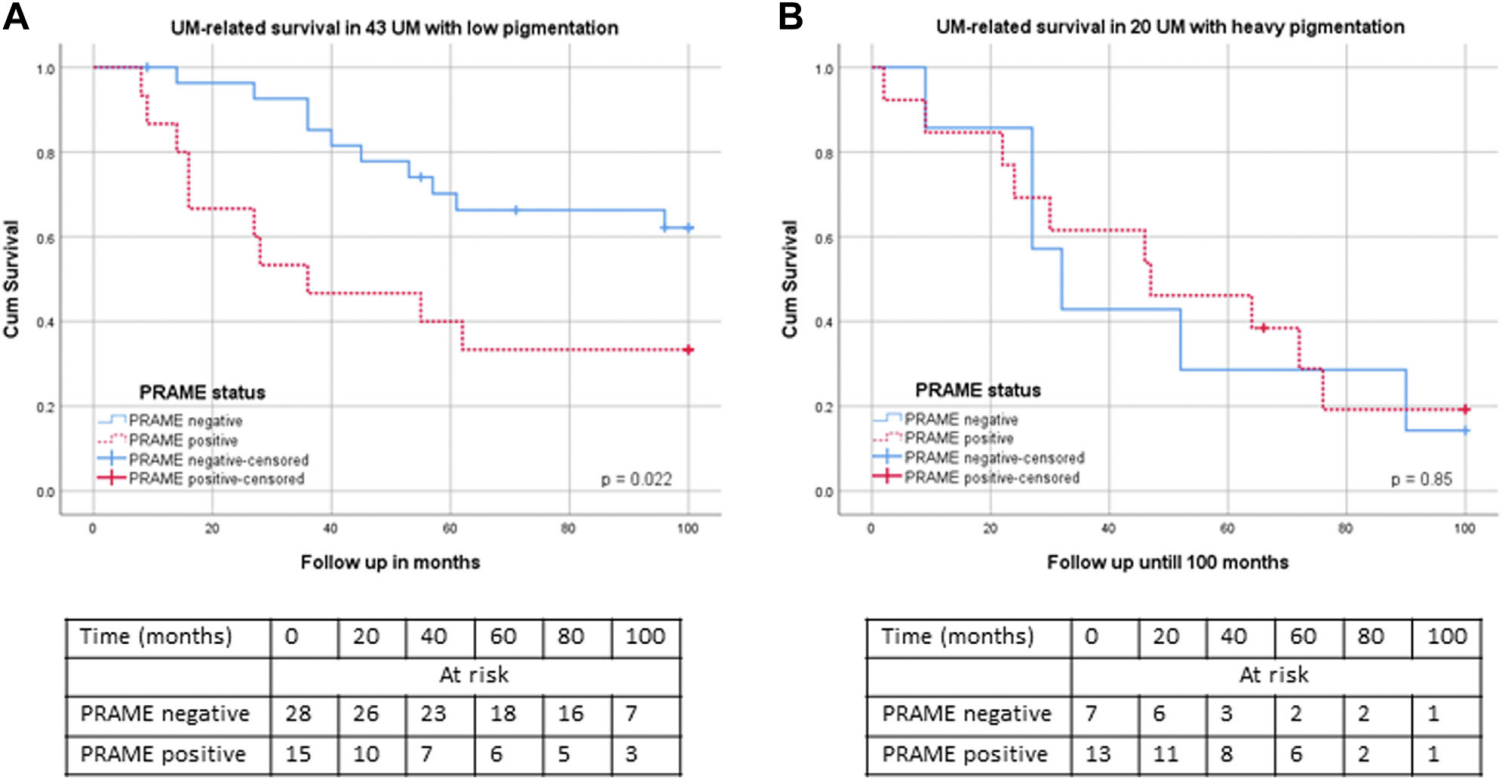
Survival in patients with UM with different PRAME expression in low pigmentation and heavy pigmentation groups. **A,** Uveal melanoma–related survival in 43 patients with low tumor pigmentation (*P* = 0.02). **B,** Uveal melanoma–related survival in 20 patients with heavy tumor pigmentation (*P* = 0.85). PRAME = preferentially expressed antigen in melanoma; UM = uveal melanoma.

We previously reported that the degree of pigmentation of the tumor influenced the relationship between chromosome 3 and survival; loss of one chromosome 3 or gain of copies of 8q had a much larger effect on survival in patients with a light tumor than in those with a heavily pigmented tumor.^16^ We now analyzed the same for *PRAME*, a potential prognostic marker, in a subset of 64 UMs with mRNA data.^19^, ^20^, ^21^ Similar to the situation with chromosome 3 and 8q, expression of *PRAME* was related to prognosis in light UM (*P* = 0.02) but not in dark tumors (*P* = 0.85) (Fig 4).

## Discussion

We studied the role of tumor pigmentation in the prognosis of UM and, in agreement with previous studies,^4^, ^5^, ^6^, ^7^, ^8^, ^9^ observed an association between heavy pigmentation and poor survival (Table 1 and Fig 2). A higher degree of pigmentation was related to larger tumor size, a more frequent involvement of the ciliary body, and a more frequent occurrence of epithelioid cells, in agreement with prior publications (Table 4). Furthermore, our results showed that darker tumors more frequently have a loss of chromosome 3/BAP1 and a gain of chromosome 8q (Table 4). The analyses performed in the D3 and M3 cohorts separately showed that chromosome 3 status is the most relevant factor related to prognosis (Fig 3), whereas in the D3 subcohort, dark tumor pigmentation showed an association with chromosome 8q gain (Table S8).

The link between chromosome 3 and tumor pigmentation is further supported by the logistic regression model presented in Table 5, which shows that both M3 and brown eye color are significant predictors of dark tumor pigmentation.

We wondered why and how M3 and tumor pigmentation could be related, and we postulate that a possible connection might be the presence of inflammation. Previous findings show that an inflammatory phenotype does not inhibit tumor growth as in other types of cancer but instead seems to be associated with the development of metastases.^22^, ^23^, ^24^, ^25^, ^26^ The number of macrophages was previously shown to be significantly correlated with the degree of tumor pigmentation, both by the Collaborative Ocular Melanoma Study analyzing choroidal melanomas and by Mäkitie et al in choroidal and ciliary body melanomas.^7^^,^^27^ The presence of lymphocytes and macrophages is associated with monosomy of chromosome 3 and carries a bad prognosis.^28^, ^29^, ^30^ Immunoregulatory forkhead box P3 (FOXp3)-positive cells were especially present in UM with M3 and with this inflammatory phenotype.^24^ More recently, a study comparing BAP1-positive and BAP1-negative tumors reported an upregulation of several immunosuppressive genes in BAP1-negative tumors, thus suggesting that the infiltrate of tumors with BAP1 loss has a regulatory and immunosuppressive T-cell phenotype.^31^ Moreover, Durante et al^32^ analyzed 8 primary UMs and 3 metastases with single-cell RNA sequencing (scRNAseq) and showed that CD8+ T cells within the tumor express the immune checkpoint lymphocyte-activation gene 3 (LAG3), which may contribute to creating an immunosuppressive environment.

We recently published^16^ that the amount of infiltrating macrophages is related both to pigmentation and chromosome 3 status; the group with the least number of macrophages consisted of lightly pigmented tumors with disomy of chromosome 3.

It seems paradoxical that pigmented UMs have an increased immune infiltrate but show more UM-related death. It is well possible that specifically the presence of pigment helps to reduce the activity of antitumor T cells; in support of an immunosuppressive property of UM cells themselves, Gezgin et al^33^ recently showed that a more successful expansion of UM-reactive tumor infiltrating lymphocytes can be achieved when the lymphocytes are separated from the tumor environment early, compared with direct culture of tumor tissue or mononuclear cell enrichment.

Evidence from cutaneous melanoma similarly suggests that increased tumor pigmentation influences tumor behavior. Brożyna et al reported that patients with amelanotic cutaneous melanomas showed better survival than patients with melanotic melanomas and that the presence of melanin in melanoma cells decreased the outcome of radiotherapy.^34^^,^^35^ In line with these findings, Slominski et al^36^ reported that pigmented cutaneous melanoma cells were more resistant to cyclophosphamide and to IL2-stimulated peripheral blood lymphocytes than unpigmented cells. They also showed that the melanogenesis intermediate L-dihydroxyphenylalanine (L-DOPA) inhibited proliferation, progression through the cell cycle, and function of lymphocytes. These results suggest that melanocytes or pigment-related molecules influence tumor behavior in cutaneous melanoma, perhaps through an effect on the immune system. Although normal cutaneous melanocytes are best known for their ability to produce melanin and to protect DNA from ultraviolet light damage, they can respond to external signals such as cytokine mediators and hormones and are able to secrete a wide range of cytokines and signaling molecules with neuroendocrine and hormonelike properties.^37^, ^38^, ^39^, ^40^, ^41^ Expression of HLA class II molecules has been reported in normal skin melanocytes upon stimulation with interferon γ.^42^, ^43^, ^44^

Melanocytes have also been shown to interact with components of the innate immunity; not only cutaneous but also choroidal melanocytes express Toll-like receptors (TLRs)^45^^,^^46^ and are able to produce and secrete cytokines and chemokines at low levels at baseline and at higher levels after stimulation with TLR agonists or with proinflammatory cytokines.^45^^,^^47^, ^48^, ^49^, ^50^ Specifically, Cioanca et al^46^ showed that human choroidal melanocytes express TLR1-6 and release more CCL2 and IL8 upon stimulation with TLR agonists. In addition, the relationship between skin melanocytes and inflammation may be influenced by the level of pigmentation. Tam et al^50^ looked at cytokine production by dark and light melanocytes upon lipopolysaccharide (LPS) stimulation. When lightly pigmented melanocytes were stimulated, they produced more proinflammatory cytokines, such as CCL2, IL6, and tumor necrosis factor-α (TNFα), than dark melanocytes.

These considerations suggest that the presence of inflammation may indeed link chromosome 3 status, inflammation, and pigmentation and that targeting the pigmentation process may help in increasing the susceptibility of melanomas to immunotherapies.

A further element to consider is the link between eye color and the degree of tumor pigmentation that was reported by Regan et al and in a previous study from our center.^8^^,^^16^ Eye color showed strong association with tumor pigmentation in D3 tumors, as well as M3 tumors (Table 6). This finding raises the possibility that tumor pigmentation might be partly influenced by the patient’s genetic background and the intrinsic level of eye pigmentation, which is determined by genes that regulate the amount and type of melanin present in melanosomes (expressed as the ratio of eumelanin and pheomelanin).^51^ Eye color is largely determined by SNPs in specific genes, such as OCA2 melanosomal transmembrane protein (*OCA2)*, HECT and RLD domain containing E3 ubiquitin protein ligase 2 (*HERC2)*, interferon regulatory factor 4 (*IRF4)*, and tyrosinase (*TYR)*,^52^, ^53^, ^54^, ^55^, ^56^ some of which have shown an association with the risk to develop UM; specific SNPs at rs12913832, rs1129038, and rs916977 in the HERC2/OCA2 locus have been associated with a lower risk of developing UM, whereas a specific SNP at rs12203592 in the IRF4 locus was associated with a higher risk of UM.^57^ Recently, Mobuchon et al^58^ hypothesized that the patient’s genes that determine eye color influence which chromosome changes/mutations occur; based on their observation that in a cohort of 972 patients with European ancestry, one of the *IRF4* SNPs (rs12203592) was a significant risk factor only in the D3 UM population, whereas an SNP on the *HERC2* locus (rs12913832) was a significant risk factor only in the M3 UM population. However, when we compare tumor chromosome aberrations in UM from individuals with different eye colors in our Leiden cohort, we do not see differences in the percentages of M3 tumors between different eye colors, with M3 being present in 56% of blue eyes, 47% of green eyes, and 57% of brown eyes (*P* = 0.45).^16^ This is in contradiction with the findings of Mobuchon. However, an important difference between the Netherlands and France that needs to be considered is that in the Netherlands, the majority of people have blue eyes. In our current study, we see that across all 3 eye color groups, darker tumors invariably contain a higher proportion of M3 cases than light tumors. Taken together, our data suggest that both chromosome 3 and 8q status and the patient’s genetic pigmentation may cooperate in determining the degree of tumor pigmentation. Further studies are needed to confirm these observations and define the specific role of each of these factors.

A limitation of this study is that we used macroscopic pigmentation, which may be influenced by several factors other than the melanin content of UM cells, such as the presence of necrosis or pigment-laden macrophages. Moreover, because these cases were collected over almost 40 years, there might be interobserver variability in pigmentation grading. However, the use of a predefined 4-point scale developed in 1984 should decrease the variability in subjective evaluation. One should also keep in mind that the clinical observations reported in this manuscript are associations and that further functional studies would be required to determine how M3 or chromosome 8q gain causes increased melanocyte pigmentation at the cellular level.

We confirm that dark tumors carry a worse prognosis than light tumors and report a significant association between dark tumor pigmentation and negative genetic prognostic factors: monosomy of chromosome 3, loss of BAP1 expression, and gain of chromosome 8q. Tumor pigmentation is not an independent prognostic factor when chromosome status is taken into account but does influence the prognostic potential of genetic changes in the tumors. Further studies are needed to determine whether the genetical eye color and therefore the type of melanin (pheo vs. eumelanin) in the tumor influence the type of chromosome change/mutation that occurs. We also propose that both tumor pigmentation and chromosome 3 status might determine the inflammatory phenotype of UM, hence contributing to the patient’s prognosis. Therefore, respective roles of chromosome 3 status/BAP1 status and pigmentation in the development of an immune response against the tumor should be explored further.
