# Supplementary material for: Increased Histological Tumor Pigmentation in Uveal Melanoma Is Related to Eye Color and Loss of Chromosome 3/BAP1
Source: Ophthalmol Sci. 2023 Mar 11;3(3):100297. doi: 10.1016/j.xops.2023.100297 (PMC10182323; doi:10.1016/j.xops.2023.100297)

Supplemental Figure 1: examples of UM with different pigmentation levels. A: unpigmented, group 1; B: lightly pigmented, group 2; C: moderately pigmented, group 3; D: heavily pigmented, group 4.

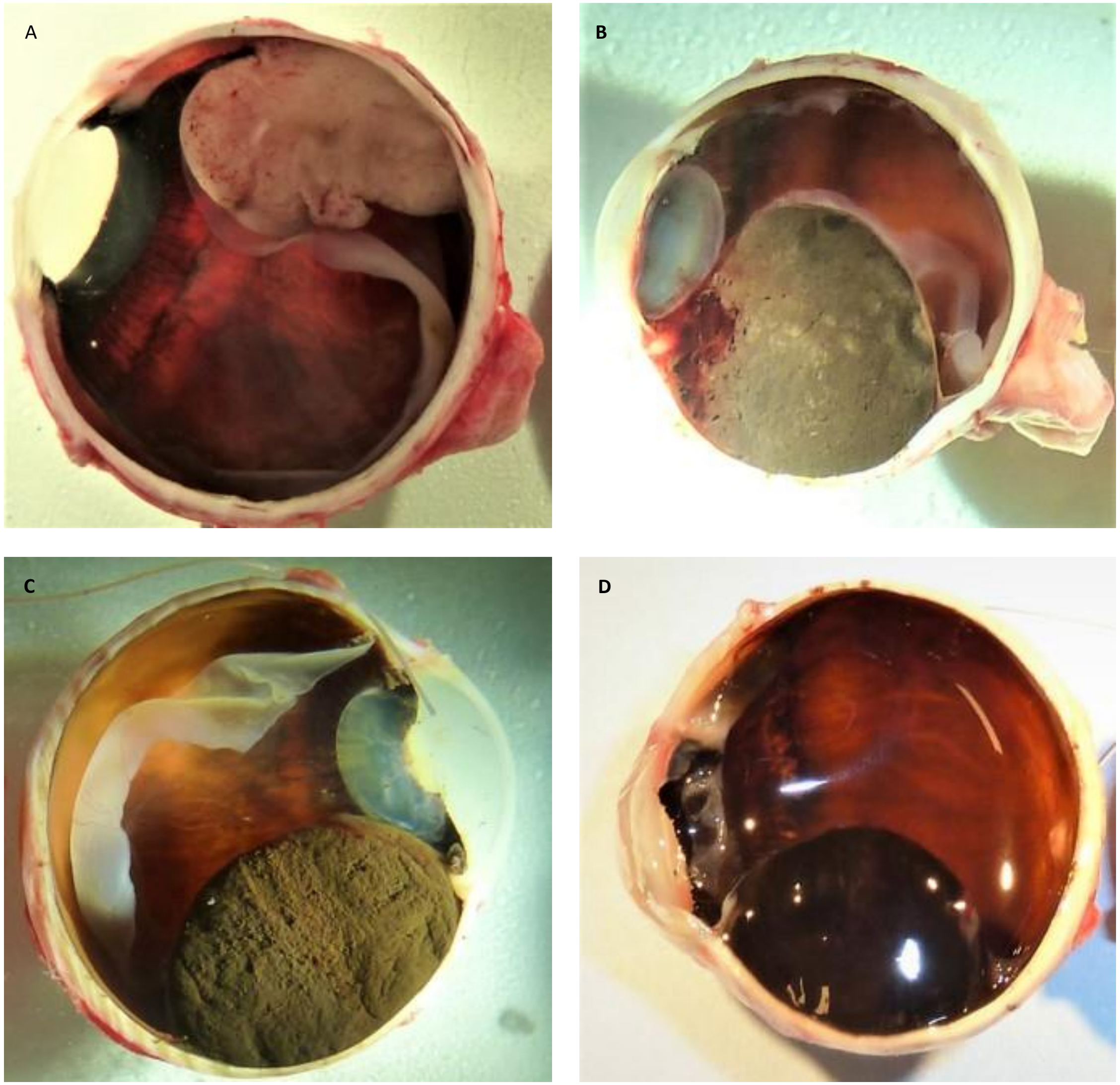

Supplement: Figure S1 [file mmc1.pdf]
