# Supplementary material for: Increased Histological Tumor Pigmentation in Uveal Melanoma Is Related to Eye Color and Loss of Chromosome 3/BAP1
Source: Ophthalmol Sci. 2023 Mar 11;3(3):100297. doi: 10.1016/j.xops.2023.100297 (PMC10182323; doi:10.1016/j.xops.2023.100297)
Supplement: Table S3 [file mmc2.pdf]

**Supplemental Table 2. Cox regression for effect of macroscopic histological pigmentation 2 groups)* on UM-related survival, correcting for age and sex in 1054 UM patients.**				
	Wald	*p* value	HR	C.I.
Dark vs light pigmentation‡*	24.361	<0.001	1.757	1.405-2.198

‡: adjusted for age and sex
*: light = unpigmented + low pigmentation; dark = moderate pigmentation + heavy pigmentation
HR: hazard ratio
C.I.: confidence interval
