# Supplementary material for: Increased Histological Tumor Pigmentation in Uveal Melanoma Is Related to Eye Color and Loss of Chromosome 3/BAP1
Source: Ophthalmol Sci. 2023 Mar 11;3(3):100297. doi: 10.1016/j.xops.2023.100297 (PMC10182323; doi:10.1016/j.xops.2023.100297)
Supplement: Table S4 [file mmc3.pdf]

**Supplemental Table 3: Cox regression for effect of pigmentation (4 groups) on survival, correcting for age and sex**				
	Wald	*p* value	HR	C.I.
Pigmentation‡	31.301	**<0.001**		
Unpigmented	13.862	**<0.001**	**0.183**	**0.075-0.447**
Low pigmentation	21.555	**<0.001**	**0.561**	**0.440-0.716**
Moderate pigment†	/	**/**	**/**	**/**
Heavy pigmentation	2.906	0.088	0.760	0.554-1.042

‡: adjusted for age and sex
†: reference group
