# Supplementary material for: Increased Histological Tumor Pigmentation in Uveal Melanoma Is Related to Eye Color and Loss of Chromosome 3/BAP1
Source: Ophthalmol Sci. 2023 Mar 11;3(3):100297. doi: 10.1016/j.xops.2023.100297 (PMC10182323; doi:10.1016/j.xops.2023.100297)
Supplement: Table S8 [file mmc4.pdf]

Supplemental Table 7. Comparison between clinical and histopathological features and tumour pigmentation (2 groups)† in patients with disomy 3 UM.

Feature	Light (130)[d]	Dark (84)[d]	*p* value[g]
**Gender**			0.73 [a]
**Male (133)**	82 (63%)	51 (61%)	
**Female (81)**	48 (37%)	33 (39%)	
**Age at enucleation (years)[e]**	62.28 (12.8-90.5)	65.57 (30.9-89.6)	0.06 [b]
**Median Follow up (months)[e]**	62.06 (0.4-252.8)	48.07 (0.2-214.7)	0.31 [b]
**Largest Basal Diameter[e]**	11 (2-22)	12 (3-19)	0.22 [b]
**Thickness[e]**	6 (0.5-12)	6 (1-15)	0.23 [b]
**Cell type**			0.010 [a]
**Spindle (65)**	48 (37%)	17 (20%)	
**Epithelioid or mixed (149)**	82 (63%)	67 (80%)	
**Ciliary body involvement**			0.029 [a]
**No (153)**	100 (77%)	53 (63%)	
**Yes (61)**	30 (23%)	31 (37%)	
**Scleral ingrowth**			
**None/superficial (149)**	86 (67%)	63 (75%)	0.22 [a]
**Deep/total (63)**	42 (33%)	21 (25%)	
**AJCC**			0.045 [a]
**I-IIB (177)**	113 (88%)	64 (77%)	
**IIIA-IIIC (35)**	16 (12%)	19 (23%)	
**8q status**			**0.003 [a]**
**Normal (156)**	104 (85%)	52 (68%)	
**Gain (43)**	18 (15%)	25 (33%)	
**BAP1 expression**			0.12 [f]
**BAP1 positive (58)**	42 (86%)	16 (70%)	
**BAP1 negative (14)**	7 (14%)	7 (30%)	

a: Pearson's χ2 test

b: Mann-Whitney U test

c: Percentages are rounded and may not total 100

d: Percentages were calculated excluding missing data

e: Median (min – max)

f: Fisher's exact test

g: α after Bonferroni correction: 0.005

†: light = unpigmented + low pigmentation; dark = moderate pigmentation + heavy pigmentation
