# Supplementary material for: Increased Histological Tumor Pigmentation in Uveal Melanoma Is Related to Eye Color and Loss of Chromosome 3/BAP1
Source: Ophthalmol Sci. 2023 Mar 11;3(3):100297. doi: 10.1016/j.xops.2023.100297 (PMC10182323; doi:10.1016/j.xops.2023.100297)
Supplement: Table S9 [file mmc5.pdf]

Supplemental Table 8: Comparison between clinical and histopathological features and tumour pigmentation (2 groups)† in patients with monosomy 3 UM.

Feature	Light (105)[d]	Dark (157)[d]	*p* value[g]
**Gender**			0.10 [a]
**Male (136)**	48 (46%)	88 (56%)	
**Female (126)**	57 (54%)	69 (44%)	
**Age at enucleation (years)[e]**	63.87 (25.8-88.3)	68.37 (30.5-93.4)	0.039 [b]
**Median Follow up (months)[e]**	30.39 (0.5-219.4)	24.77 (0.1-174.4)	0.10 [b]
**Largest Basal Diameter[e]**	13 (6-30)	13 (2-20)	0.98 [b]
**Thickness[e]**	7 (1-15)	8 (1-17)	0.96 [b]
**Cell type**			0.20 [a]
**Spindle (43)**	21 (20%)	22 (14%)	
**Epithelioid or mixed (219)**	84 (80%)	135 (86%)	
**Ciliary body involvement**			0.34 [a]
**No (113)**	49 (47%)	64 (41%)	
**Yes (149)**	56 (53%)	93 (59%)	
**Scleral ingrowth**			0.51 [a]
**None/superficial (172)**	71 (68%)	101 (64%)	
**Deep/total (89)**	33 (32%)	56 (36%)	
**AJCC**			0.11 [a]
**I-IIB (156)**	67 (67%)	89 (57%)	
**IIIA-IIIC (100)**	33 (33%)	67 (43%)	
**8q status**			0.54 [a]
**Normal (53)**	24 (25%)	29 (21%)	
**Gain (180)**	73 (76%)	107 (79%)	
**BAP1 expression**			0.24 [f]
**BAP1 positive (14)**	7 (17%)	7 (9%)	
**BAP1 negative (103)**	34 (83%)	69 (91%)	

a: Pearson's $\chi 2$ test

b: Mann-Whitney U test

c: Percentages are rounded and may not total 100

d: Percentages were calculated excluding missing data

e: Median (min – max)

f: Fisher's exact test

g: α after Bonferroni correction: 0.005

†: light = unpigmented + low pigmentation; dark = moderate pigmentation + heavy pigmentation
